# Development and Validation of a Nomogram for Balloon Pulmonary Angioplasty-Related Complications in Patients with Chronic Thromboembolic Pulmonary Hypertension

**DOI:** 10.31083/j.rcm2403072

**Published:** 2023-02-28

**Authors:** Xin Li, Yi Zhang, Qi Jin, Qin Luo, Qing Zhao, Tao Yang, Qixian Zeng, Anqi Duan, Zhihua Huang, Meixi Hu, Sicheng Zhang, Luyang Gao, Changming Xiong, Zhihui Zhao, Zhihong Liu

**Affiliations:** ^1^Center for Respiratory and Pulmonary Vascular Diseases, Fuwai Hospital, National Center for Cardiovascular Diseases, National Clinical Research Center for Cardiovascular Diseases, Chinese Academy of Medical Sciences and Peking Union Medical College, 100037 Beijing, China; ^2^Department of Cardiology, Zhongshan Hospital, Shanghai Institute of Cardiovascular Diseases, Fudan University, 200032 Shanghai, China

**Keywords:** chronic thromboembolic pulmonary hypertension, balloon pulmonary angioplasty, right heart catheterization, complications

## Abstract

**Background:**

Balloon pulmonary angioplasty (BPA)-related complications are not uncommon and could contribute to perioperative mortality. However, there is a lack of a prediction model for BPA-related complications.

**Methods:**

Data from consecutive patients diagnosed with chronic thromboembolic pulmonary hypertension (CTEPH) who underwent BPA were retrospectively analyzed. The primary outcome was BPA-related complications. The secondary outcomes were mortality and hemodynamics after BPA.

**Results:**

A total of 207 patients with 614 BPA sessions were included. Complications occurred during 63 sessions (10.26%) in 49 patients. Hemoptysis or hemosputum (6.51%) was the most common complication, whereas pulmonary reperfusion edema was rare (0.49%). Multivariable logistic regression identified that disease duration, mean pulmonary arterial pressure (mPAP) and the proportion of occlusion lesions were correlated with BPA complications. A nomogram was constructed accordingly, which had the highest area under curve (0.703) and was superior to previously reported predictors [nomogram vs. mPAP, net reclassification index (95% confidence interval (CI)), 0.215 (0.002, 0.427), *p* = 0.047; integrated discrimination index (95% CI), 0.059 (0.010, 0.109), *p* = 0.018]. The nomogram was found to be accurate based on validation and calibration (slope 0.978, Bier score 0.163). After adjusting for the number of BPA sessions in multivariable linear regression, the occurrence of complications was not associated with hemodynamic improvement after BPA. The 3-year survival was also comparable between patients with and without complications (98.0% vs. 94.8%, log-rank *p* = 0.503).

**Conclusions:**

The nomogram, comprising mPAP, the proportion of occlusion lesions and disease duration, could better predict BPA-related complications than previously reported single parameters. Distinctively, the occurrence of complications did not impair the beneficial impact of BPA on hemodynamics and survival. The occurrence of complications should not discourage patients from continuing BPA sessions.

## 1. Introduction

Chronic thromboembolic pulmonary hypertension (CTEPH) is a progressive disease characterized by organized thrombi obstruction in major pulmonary arteries [1], along with peripheral microvasculopathy in nonoccluded areas, which could lead to right heart decompensation and death [2]. Pulmonary endarterectomy (PEA) is regarded as the standard treatment for CTEPH and can significantly improve clinical presentation and survival [3], whereas approximately 40% of patients are not eligible for PEA due to distal lesions or comorbidities [4]. Balloon pulmonary angioplasty (BPA) is a promising treatment for patients with inoperable CTEPH or patients suffering from persistent pulmonary hypertension (PH) after PEA; this treatment could significantly improve hemodynamics, exercise capacity and survival [5].

Despite the promising efficacy of BPA, complications are commonly observed, with the reported prevalence ranging from 6% [6] to 15.5% [7]. Severe complications not only increase the length of hospital stay [8] but also contribute to perioperative mortality. Identifying predictors of BPA-related complications is crucial to guiding BPA performance. Previous studies have identified that high baseline mean pulmonary arterial pressure (mPAP) [9], pulmonary vascular resistance (PVR) [8], and N-terminal pro-brain natriuretic peptide (NT-proBNP) [10] are associated with an increased risk of BPA complications. However, these studies are limited to a single parameter, and there is a lack of a prediction model of BPA-related complications. Moreover, the impact of complications on hemodynamic outcome and long-term survival remains elusive. Herein, we aimed to develop and validate a nomogram with multiple variables to predict the complications of BPA. Furthermore, we aimed to evaluate whether the occurrence of complications would impair hemodynamic outcome and long-term survival after BPA.

## 2. Materials and Methods

### 2.1 Study Design and Participants

Consecutive patients diagnosed with CTEPH according to guidelines [11] who underwent BPA between May 2018 and June 2022 in Fuwai Hospital, Chinese Academy of Medical Sciences (Beijing, China) were included in the study. The Ethics Committee of Fuwai Hospital gave approval to the current study protocol (Approval NO: 2020-1275), and the current study was in compliance with the ISHLT Ethics statement. Informed consent was obtained from each patient. The diagnosis of CTEPH was based on the 2015 ESC/ERS guidelines [11], and the eligibility for BPA was based on a multidisciplinary discussion involving pulmonary vascular specialists, PEA surgeons and interventional specialists. Demographics, exercise capacity, laboratory tests and echocardiography were evaluated prior to and after every BPA session.

### 2.2 Right Heart Catheterization (RHC) and BPA Procedural Details

The RHC and BPA procedural details were in accordance with our previous description and are detailed in the **Supplementary Material **[5]. Before each BPA session, RHC was performed to obtain the hemodynamic parameters. Subsequently, BPA was performed with selective pulmonary angiography and lung ventilation/perfusion scintigraphy as a reference. Hemodynamics were measured again after each BPA session. According to previous studies [6, 7], pulmonary vessel lesions were classified into occlusive lesions (subtotal occlusion and total occlusion) and nonocclusive lesions (web lesions, ring-like stenosis and tortuous lesions) [6].

### 2.3 Cardiopulmonary Exercise Testing

Symptom-limited cardiopulmonary exercise testing was performed on a cycle ergometer (COSMED Quark cardiopulmonary exercise testing system) [12]. Initially, patients rested for three minutes and then pedaled without workload for three minutes. Subsequently, the work rate increased gradually according to individual expectations until the maximum exercise tolerance. During the exercise, minute ventilation, oxygen uptake (${\mathrm{VO}}_{2}$) and carbon dioxide output were measured with breath-by-breath gas analysis. Peak ${\mathrm{VO}}_{2}$ was determined by averaging the highest 30-second value of ${\mathrm{VO}}_{2}$ during the last minute of exercise. The minute ventilation versus carbon dioxide output slope was determined by the linear relationship between minute ventilation and carbon dioxide output during the entire exercise period.

### 2.4 Outcome

The primary outcome was the occurrence of BPA-related complications, which was defined in line with previous studies, including hemoptysis or hemosputum, pulmonary artery dissection, access site complication, vascular injury, and pulmonary reperfusion injury [6].

There were two secondary outcomes. The first was all-cause mortality. After discharge, patients were followed up via telephone, outpatient and/or inpatient visits regularly. The survival time was calculated from baseline to the occurrence of death or the censored date, namely, September 2022. The other secondary outcome was hemodynamics at the latest reevaluation RHC or the hemodynamic measurement prior to the latest BPA.

### 2.5 Statistical Analysis

The distribution of variables was examined by the Kolmogorov-Smirnov test. Accordingly, normally distributed continuous parameters are presented as the mean ± standard deviation and were examined by independent-sample *t*tests. Abnormally distributed continuous parameters are presented as the median (interquartile range) and were examined by the Mann–Whitney *U* test. Categorical variables are given as counts (percentages), and the* chi*-square test with or without continuity correction or Fisher’s exact test was used to compare differences between groups. Univariable logistic regressions were used to explore potential parameters correlated with BPA-related complications (*p *< 0.05 in univariable logistic regressions). Subsequently, variables of statistical significance were included in multivariable logistic regression with the backward method. Receiver operator characteristic curve (ROC) analyses and area under the curve (AUC) were applied to compare the prediction ability of the models. Decision curve analysis, net reclassification index and integrated discrimination index were used to compare the clinical usefulness and net benefit of different models. A nomogram was constructed based on the final model (R package rms, https://CRAN.R-project.org/package=rms). A calibration curve was performed to evaluate the agreement between the predicted and observed risks. Internal validation was performed for the final model using bootstrapping with 1000 repetitions (R package rms, https://CRAN.R-project.org/package=rms). The Kaplan–Meier method with the log-rank test was used to compare survival between patients with or without complications. No missing values had to be replaced. Statistical significance was set at* p *< 0.05. SPSS 26.0 (IBM SPSS Corp., Armonk, NY, USA), R (version 4.0.5, R Foundation for Statistical Computing, Vienna, Austria) and Prism GraphPad 9 (GraphPad Software, LA Jolla, CA, USA) were used for statistical analyses. Z.L. had full access to all the data in the study and took responsibility for its integrity and data analysis.

## 3. Results

### 3.1 Baseline Demographics of Included Patients

A total of 207 patients underwent 614 BPA sessions from May 2018 to June 2022. The mean age of the included patients was 59.47 ± 10.86 years old, and 113 (54.6%) were female (Table 1). BPA-related complications occurred during 63 sessions (10.26% of all 614 sessions) in 49 patients (23.67% of all 207 patients) (Table 2).

**Table 1.** **Clinical features of patients with complications and without complications at baseline**.

Variables		Total (n = 207)	Without complications	With complications	*p* value
			(n = 158)	(n = 49)	
Age, years		59.47 ± 10.86	59.08 ± 11.40	60.71 ± 8.90	0.563
Female, n (%)		113 (54.59)	80 (50.63)	33 (67.35)	**0.040**
BMI, kg/$m^{2}$		23.94 ± 3.30	23.87 ± 3.32	24.15 ± 3.23	0.597
WHO FC					0.083
	I or II, n (%)	72 (34.78)	60 (37.97)	12 (24.49)	
	III or IV, n (%)	135 (65.22)	98 (62.03)	37 (75.51)	
Disease duration, years		3.00 (1.00, 7.00)	3.00 (1.00, 6.00)	5.00 (1.00, 10.00)	0.063
NT-proBNP, ng/L		780.00 (202.30, 1706.00)	686.00 (176.90, 1694.75)	1055.00 (425.50, 2020.00)	**0.042**
6MWD, m		354.60 ± 111.98	364.54 ± 111.34	325.18 ± 109.76	**0.028**
Targeted therapy at baseline					0.199
	None	57 (27.54)	40 (25.32)	17 (34.69)	
	Mono-therapy/Combination	150 (72.46)	118 (74.68)	32 (65.31)	
Echocardiography					
	LA, mm	34.60 ± 5.85	34.42 ± 5.26	35.20 ± 7.48	0.413
	RVED/LVED	0.84 ± 0.25	0.83 ± 0.26	0.88 ± 0.24	0.180
	EF, %	65.67 ± 6.46	65.54 ± 6.58	66.08 ± 6.13	0.612
	TRV, m/s	4.28 ± 0.71	4.21 ± 0.72	4.50 ± 0.63	**0.012**
Hemodynamics					
	$S_{\text{v}}$$O_{2}$, %	66.75 ± 6.83	67.09 ± 7.08	65.63 ± 5.86	0.191
	mRAP, mmHg	7.84 ± 3.70	7.54 ± 3.60	8.82 ± 3.89	0.050
	sPAP, mmHg	87.92 ± 22.24	85.83 ± 22.01	94.67 ± 21.83	**0.015**
	dPAP, mmHg	30.30 ± 8.24	29.40 ± 8.02	33.22 ± 8.32	**0.005**
	mPAP, mmHg	48.78 ± 11.42	47.32 ± 10.95	53.51 ± 11.71	**0.001**
	PAWP, mmHg	10.28 ± 2.40	10.13 ± 2.30	10.78 ± 2.66	0.338
	Cardiac index, L/(min·$m^{2}$)	2.71 ± 0.64	2.76 ± 0.65	2.57 ± 0.61	0.071
	PVR, wood units	9.47 ± 4.57	9.08 ± 4.56	10.75 ± 4.43	**0.011**
	Occlusion lesions, ${\%}^{\$}$	15.79 (5.00, 33.10)	10.82 (0.00, 31.58)	19.05 (10.53, 43.65)	< **0.001**
CPET					
	DLCO, % predicted	61.27 ± 16.14	61.50 ± 16.40	60.56 ± 15.51	0.935
	${\mathrm{FEV}}_{1}$/FVC	0.74 ± 0.08	0.75 ± 0.07	0.72 ± 0.08	0.124
	${\mathrm{VO}}_{2}$@Peak, mL/min/kg	12.22 ± 3.65	12.59 ± 3.92	11.11 ± 2.37	**0.005**
	$P_{\text{ET}}$${\mathrm{CO}}_{2}$@Peak, mmHg	24.90 ± 5.63	25.11 ± 5.73	24.29 ± 5.36	0.439
	VE/${\mathrm{VCO}}_{2}$ slope	48.51 ± 10.19	48.33 ± 10.88	49.08 ± 7.87	0.331

Data are presented as mean ± standard deviation, median (interquartile range) or number (percentage). BPA, balloon pulmonary angioplasty; BMI, body mass index; DLCO, diffusing capacity for carbon monoxide; dPAP, diastolic pulmonary arterial pressure; EF, ejection fraction; ${\mathrm{FEV}}_{1}$/FVC, forced expiratory volume in one second/forced vital capacity; LA, anteroposterior diameter of left atrium;, mPAP, mean pulmonary arterial pressure; mRAP, mean right atrial pressure; NT-proBNP, N-terminal pro-brain natriuretic peptide; PAWP, pulmonary arterial wedge pressure; $P_{\text{ET}}$${\mathrm{CO}}_{2}$, end-tidal partial pressure of carbon dioxide; PVR, pulmonary vascular resistance; RVED/LVED, right ventricular end-diastolic diameter/left ventricular end-diastolic diameter; 6MWD, 6-min walk distance; sPAP, systolic pulmonary arterial pressure; $S_{\text{v}}$$O_{2}$, mixed venous oxygen saturation; TRV, tricuspid regurgitation velocity; VE/${\mathrm{VCO}}_{2}$, minute ventilation/carbon dioxide production; ${\mathrm{VO}}_{2}$@Peak, peak oxygen consumption; WHO FC, World Health Organization functional class. Significant *p* values (*p *< 0.05) are bolded. ^$^ Occlusion lesions refer to the proportion of occlusion lesions in total pulmonary vascular lesions at baseline.

**Table 2.** **Procedure and Patient Level Analysis of BPA-related complications**.

Complications		Procedure level (n = 614)	Patient level* (n = 207)
Overall complications		63 (10.26)	49 (23.67)
	Hemoptysis or hemosputum	40 (6.51)	32 (15.46)
	Mild vascular injury	13 (2.12)	11 (5.31)
	Pulmonary artery dissection	3 (0.49)	3 (1.45)
	Access site complication	4 (0.65)	4 (1.93)
	Reperfusion injury	3 (0.49)	3 (1.45)

Data are presented as number (percentage). *Among 49 patients with complications, some patients experienced more than one type of complication. BPA, balloon pulmonary angioplasty.

### 3.2 Patients with BPA-Related Complications vs. without BPA-Related Complications

At baseline, patients with BPA-related complications had a higher proportion of females, worse cardiac function [NT-proBNP 1055.00 (425.50, 2020.00) ng/L vs. 686.00 (176.90, 1694.75) ng/L, *p* = 0.042], poorer hemodynamics (mPAP 53.51 ± 11.71 mmHg vs. 47.32 ± 10.95 mmHg, *p* = 0.001) (Table 1) and a higher proportion of occlusion lesions (19.05% vs. 10.82%, *p *< 0.001) than patients without complications.

Among the 63 complications, hemoptysis or hemosputum was most commonly observed (40, 6.51% of all BPA sessions). Pulmonary reperfusion edema occurred in three patients (0.49%), and one of the patients died (Table 2). Among 63 cases of complications, 54 cases were treated with balloon occlusion, 57 cases with continuous positive airway pressure (CPAP) and one case with extracorporeal membrane oxygenation. Among the 4 patients with access site complications, 3 patients were treated with compression bandages, and one received surgical repair.

### 3.3 Predictors of BPA-Related Complications

**Fig. 1.**
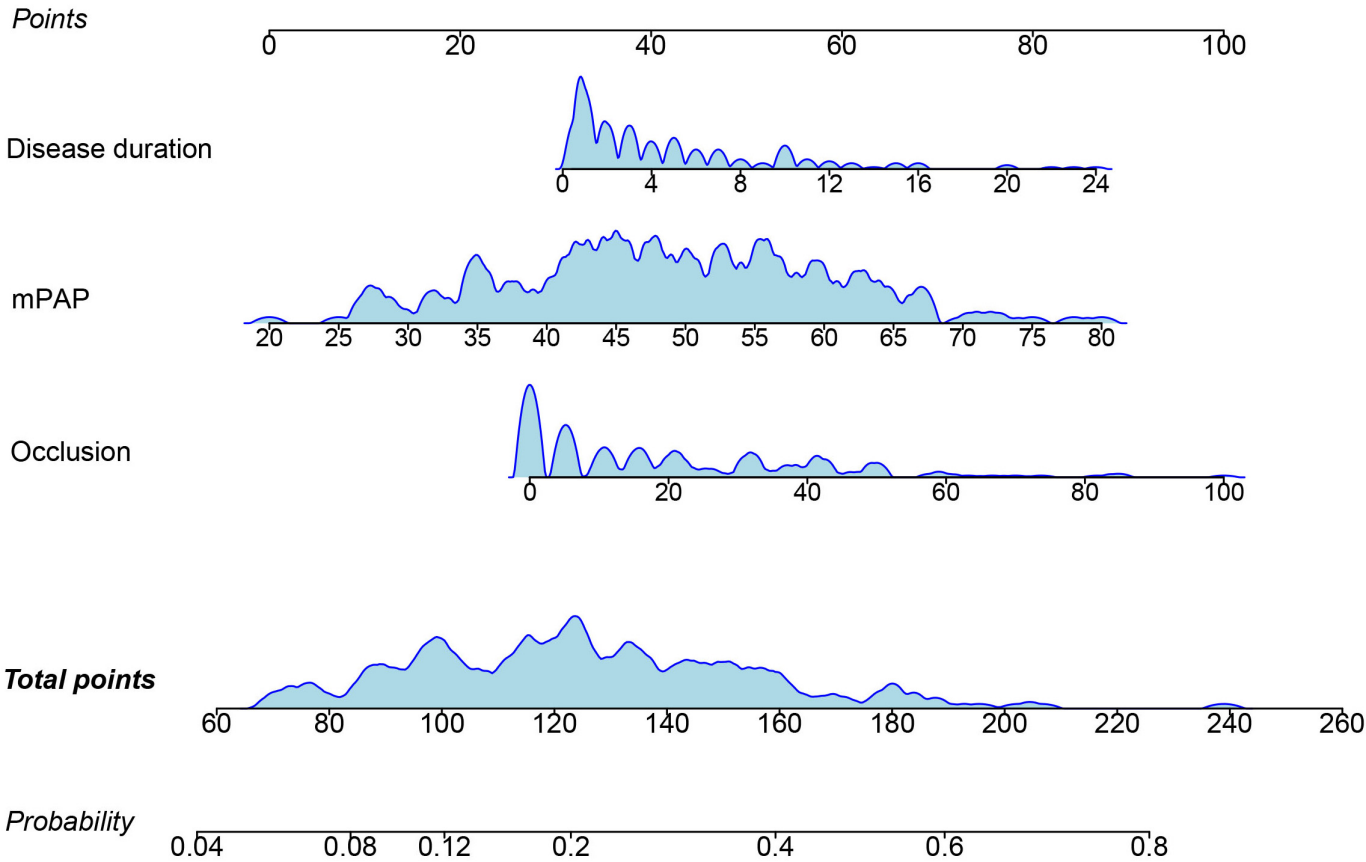
**Nomogram of BPA-related complications**. BPA, balloon pulmonary angioplasty; mPAP, mean pulmonary arterial hypertension. Occlusion refers to the proportion of occlusion lesions among total pulmonary vascular lesions at baseline.

Univariable logistic regression identified that female sex, disease duration, 6-min walk distance (6MWD), mean right atrial pressure, systolic PAP, diastolic PAP, mPAP, the proportion of occlusion lesions, PVR, NT-proBNP and ${\mathrm{VO}}_{2}$@Peak were associated with BPA-related complications (Table 3). Multivariable logistic regression with the backward method further identified that disease duration, mPAP and proportion of occlusion lesions were associated with BPA-related complications. A nomogram was constructed based on the multivariable regression. The higher the total points, the higher the risk of BPA-related complications (Fig. 1).

**Table 3.** **Logistic regression analysis for BPA complication**.

Variables	Univariable			Multivariable		
	OR	95% CI	*p*-value	OR	95% CI	*p*-value
Age	1.015	0.984–1.047	0.358			
BMI	1.027	0.932–1.131	0.596			
Female	2.011	1.025–3.944	**0.042**			
WHO FC	1.888	0.913–3.902	0.086			
Ln (NT-proBNP)	1.316	1.023–1.692	**0.032**			
6MWD	0.997	0.994–1.000	**0.035**			
Disease duration	1.095	1.028–1.167	**0.005**	1.065	0.993**–**1.142	0.077
RVED/LVED	2.297	0.676–7.801	0.183			
EF	1.013	0.964–1.064	0.611			
$S_{\text{v}}$ $O_{2}$	0.969	0.925–1.016	0.192			
mRAP	1.093	1.005–1.189	**0.037**			
sPAP	1.019	1.003–1.035	**0.016**			
dPAP	1.058	1.017–1.101	**0.005**			
mPAP	1.051	1.020–1.084	**0.001**	1.040	1.007**–**1.074	**0.016**
Cardiac index	0.624	0.373–1.045	0.073			
PVR	1.080	1.008–1.156	**0.028**			
DLCO	0.996	0.975–1.018	0.747			
${\mathrm{FEV}}_{1}$/FVC	0.028	0.001–2.762	0.127			
${\mathrm{VO}}_{2}$@Peak	0.884	0.792–0.987	**0.028**			
$P_{\text{ET}}$${\mathrm{CO}}_{2}$@Peak	0.974	0.912–1.039	0.423			
VE/${\mathrm{VCO}}_{2}$ slope	1.007	0.972–1.043	0.689			
Proportion of occlusion ${\mathrm{lesions}}^{\$}$	1.027	1.012–1.043	**0.001**	1.020	1.003–1.037	**0.021**

BPA, balloon pulmonary angioplasty; BMI, body mass index; CI, confidence interval; dPAP, diastolic pulmonary arterial pressure; DLCO, diffusing capacity for carbon monoxide; EF, ejection fraction; ${\mathrm{FEV}}_{1}$/FVC, forced expiratory volume in one second/forced vital capacity; mPAP, mean pulmonary arterial pressure; mRAP, mean right atrial pressure; NT-proBNP, N-terminal pro-brain natriuretic peptide; OR, odds ratio; $P_{\text{ET}}$${\mathrm{CO}}_{2}$, end-tidal partial pressure of carbon dioxide; PVR, pulmonary vascular resistance; RVED/ LVED, right ventricular end-diastolic diameter/left ventricular end-diastolic diameter; 6MWD, 6-min walk distance; sPAP, systolic pulmonary arterial pressure; $S_{\text{v}}$$O_{2}$, mixed venous oxygen saturation; TRV, tricuspid regurgitation velocity; VE/${\mathrm{VCO}}_{2}$, minute ventilation/carbon dioxide production; ${\mathrm{VO}}_{2}$@Peak, peak oxygen consumption; WHO FC, World Health Organization functional class; Significant *p* values (*p *< 0.05) are bolded. ^$^ Proportion of occlusion lesions refers to the proportion of occlusion lesions in total pulmonary vascular lesions at baseline.

**Fig. 2.**
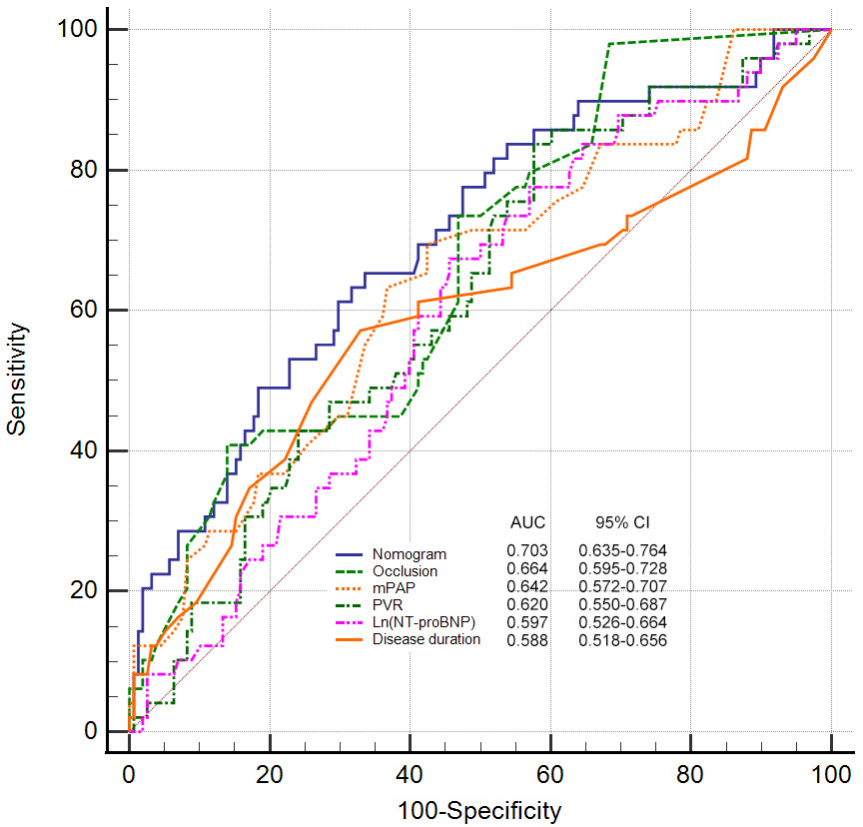
**Receiver operator characteristic curve of parameters in predicting BPA-related complications**. AUC, area under curve; BPA, Balloon pulmonary angioplasty; CI, confidence interval; NT-proBNP, N-terminal pro-brain natriuretic peptide; mPAP, mean pulmonary arterial pressure; PVR, pulmonary vascular resistance; nomogram comprising disease duration, mean pulmonary arterial pressure and proportion of occlusion lesions. Occlusion refers to the proportion of occlusion lesions among total pulmonary vascular lesions at baseline.

**Fig. 3.**
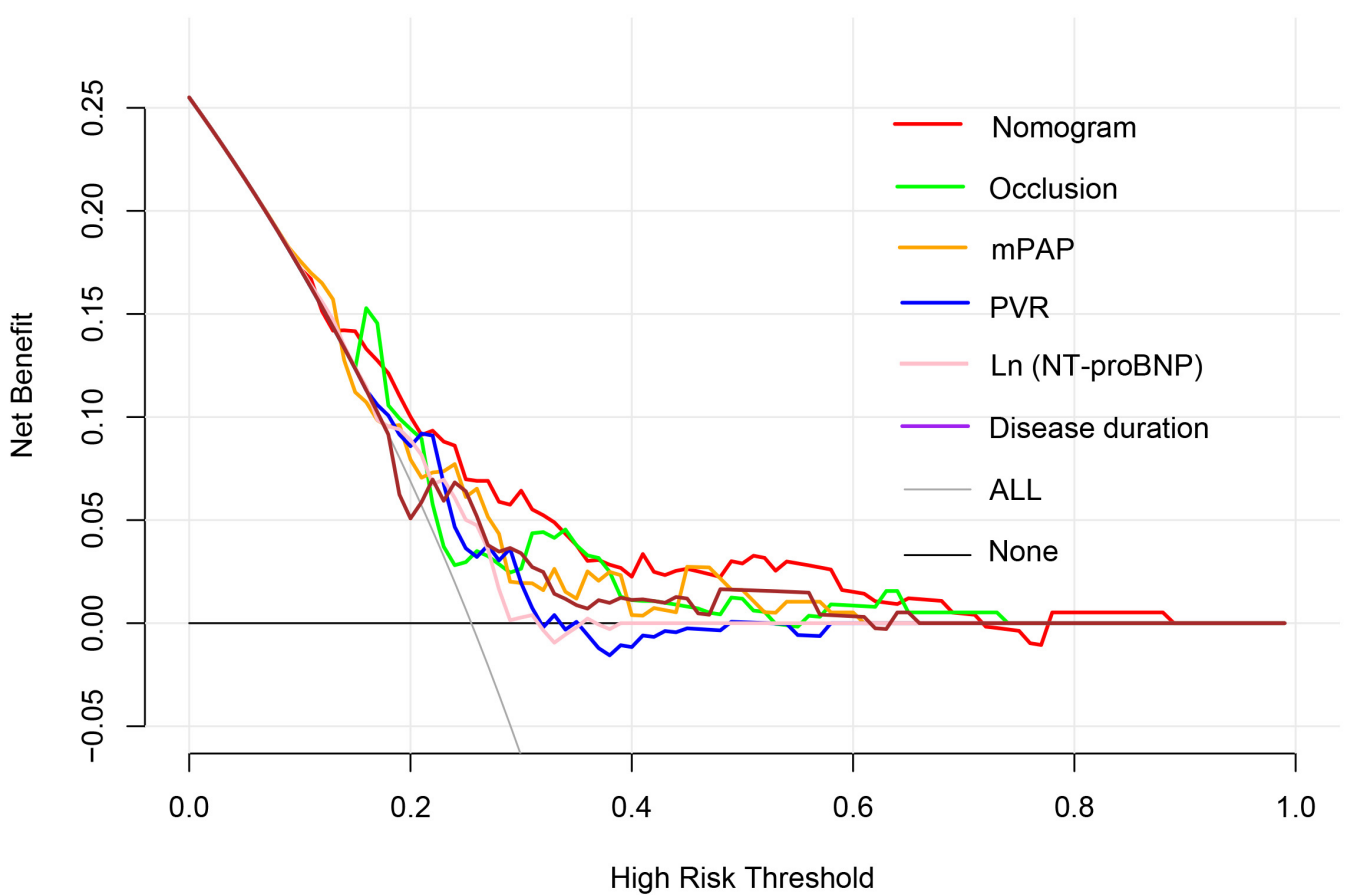
**Decision curve analysis of parameters in predicting BPA-related complications**. BPA, balloon pulmonary angioplasty; NT-proBNP, N-terminal pro-brain natriuretic peptide; mPAP, mean pulmonary arterial pressure; PVR, pulmonary vascular resistance; nomogram comprising disease duration, mean pulmonary arterial pressure and proportion of occlusion lesions. Occlusion refers to the proportion of occlusion lesions in total pulmonary vascular lesions at baseline.

**Fig. 4.**
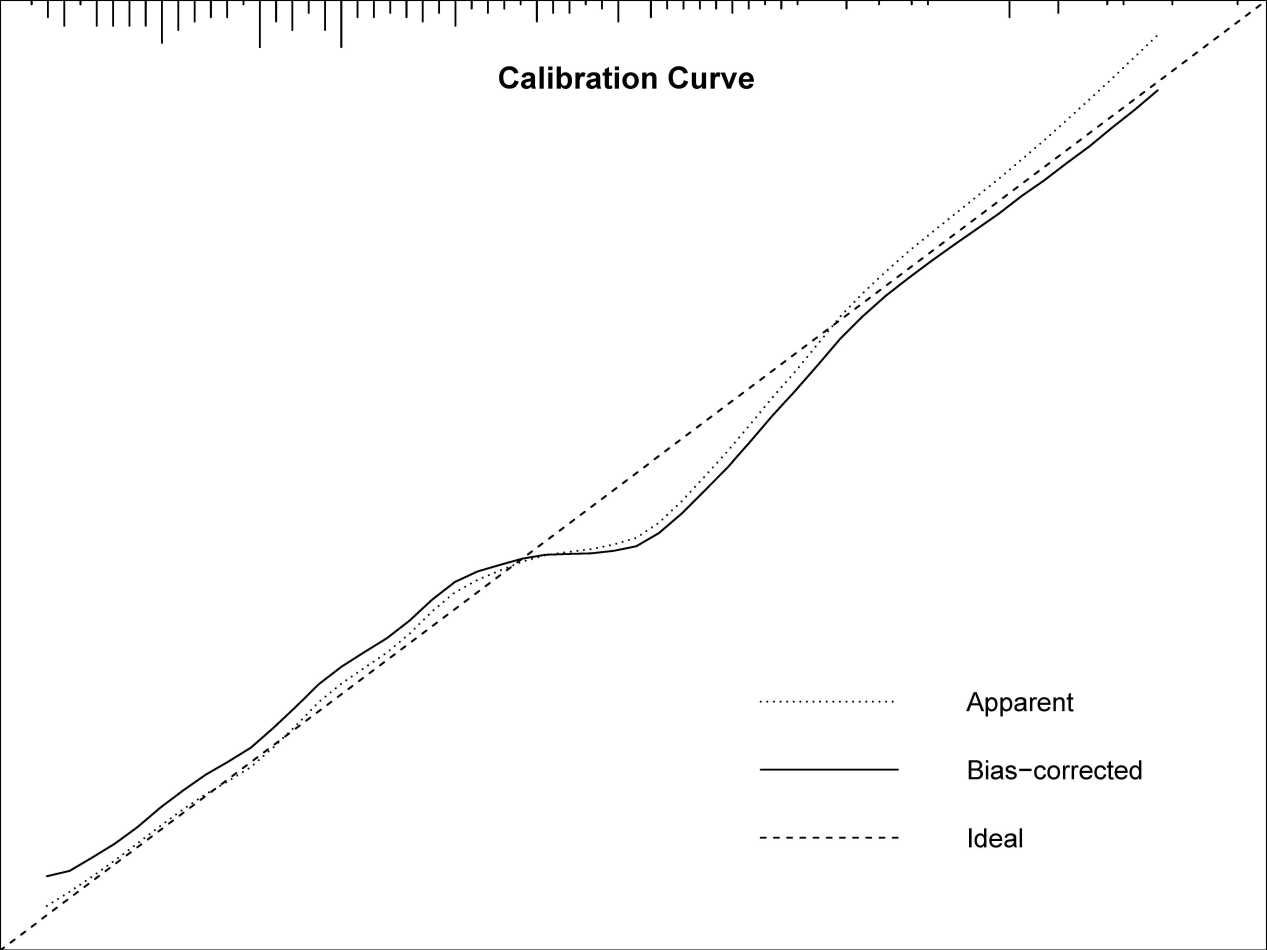
**Calibration of nomogram for predicting BPA-related complications**. BPA, balloon pulmonary angioplasty; slope of curve 0.978, Bier score of calibration 0.163.

We used ROC to compare the predictive ability of the nomogram and predictors identified in previous studies, namely, PVR, mPAP, and NT-proBNP. The nomogram had the highest AUC (AUC 0.703, 95% CI 0.635–0.764) (Fig. 2) compared to the other predictors. The clinical utility of the models was further compared by decision curve analysis (Fig. 3), which graphically illustrated the clinical utility of each model based on a continuum of potential thresholds for the risk of complications (x-axis) and the net benefit of using the model to risk stratify patients (y-axis) relative to assuming that no patient would have BPA-related complications. According to Fig. 3, the nomogram demonstrated the largest net benefit across the range of risk of BPA-related complications compared with previous predictors [nomogram vs. mPAP, net reclassification index (95% CI), 0.215 (0.002, 0.427), *p* = 0.047; integrated discrimination index (95% CI), 0.059 (0.010, 0.109), *p* = 0.018]. Internal validation by bootstrapping with 1000 samples further validated the predictive ability of the model with a concordance index of 0.68. The calibration curve illustrated the excellent agreement between the predicted and actual incidence of complications after BPA (slope 0.978, Bier score 0.163) (Fig. 4).

### 3.4 Impact of BPA-Related Complications on Hemodynamic Outcome

Among 207 patients, 160 patients had follow-up hemodynamics. Compared with patients without BPA-related complications, patients with BPA-related complications underwent more BPA sessions [3 (2, 5) vs. 2 (1, 3), *p *< 0.001] and longer time intervals from baseline to follow-up [385 (274, 658) days vs. 235 (109, 500) days, *p *< 0.001] (Table 4). Both patients with and without BPA-related complications had significant improvement in cardiac function and hemodynamics [without complications pre vs. post BPA: mPAP 48.26 ± 10.92 mmHg vs. 37.75 ± 9.10 mmHg, *p *< 0.001; with complications pre vs. post BPA: mPAP 53.22 ± 12.02 mmHg vs. 38.71 ± 11.09, *p *< 0.001]. There was a trend that patients with complications had more pronounced improvement in hemodynamics than those without complications (ΔPVR –4.37 (–6.15, –1.72) wood units vs. –2.30 (–4.94, –0.81) wood units, *p* = 0.021) (**Supplementary Table 1**), which might be attributable to more BPA sessions in patients with complications. After adjusting for BPA sessions in multivariable linear regression, the occurrence of complications was not associated with the change in hemodynamics after BPA (**Supplementary Table 2**).

**Table 4.** **Clinical features of patients with complications and without complications at follow-up**.

Variables		Without complications (n = 115)		*p* value	With complications (n = 45)		*p* value
		Before	After		Before	After	
WHO FC				< **0.001**			< **0.001**
	I or II, n (%)	42 (36.52)	94 (81.74)		11 (24.44)	36 (80.00)	
	III or IV, n (%)	73 (63.48)	21 (18.26)		34 (75.56)	9 (20.00)	
NT-proBNP, ng/L		719.00 (189.10, 1544.00)	146.00 (63.30, 366.00)	< **0.001**	1035.00 (354.00, 2020.00)	217.20 (81.35, 661.10)	< **0.001**
6MWD, m		371.43 ± 103.82	443.37 ± 94.62	< **0.001**	335.56 ± 111.17	438.95 ± 65.16	< **0.001**
Echocardiography							
	LA, mm	34.25 ± 5.58	35.09 ± 4.99	**0.022**	35.42 ± 7.70	36.38 ± 6.16	0.377
	RVED/LVED	0.82 ± 0.25	0.70 ± 0.17	< **0.001**	0.87 ± 0.24	0.69 ± 0.15	< **0.001**
	EF, %	64.31 ± 6.18	65.76 ± 6.09	0.083	65.67 ± 5.97	65.78 ± 4.19	0.906
	TRV, m/s	4.28 ± 0.60	3.83 ± 0.68	< **0.001**	4.53 ± 0.63	3.72 ± 0.72	< **0.001**
Hemodynamics							
	$S_{\text{v}}$$O_{2}$, %	67.64 ± 6.62	68.68 ± 6.00	0.085	65.67 ± 5.65	67.73 ± 6.24	**0.044**
	mRAP, mmHg	7.51 ± 3.60	7.07 ± 3.15	0.218	8.89 ± 3.98	8.09 ± 3.36	0.197
	sPAP, mmHg	88.08 ± 20.45	67.48 ± 18.68	< **0.001**	94.18 ± 21.96	67.18 ± 23.16	< **0.001**
	dPAP, mmHg	29.57 ± 7.62	23.38 ± 6.30	< **0.001**	33.20 ± 8.42	24.27 ± 7.37	< **0.001**
	mPAP, mmHg	48.26 ± 10.92	37.75 ± 9.10	< **0.001**	53.22 ± 12.02	38.71 ± 11.09	< **0.001**
	PAWP, mmHg	10.10 ± 2.12	10.42 ± 2.54	0.120	10.84 ± 2.68	11.73 ± 4.21	0.086
	Cardiac index, L/(min·$m^{2}$)	2.76 ± 0.60	3.15 ± 0.71	< **0.001**	2.57 ± 0.60	3.21 ± 0.67	< **0.001**
	PVR, wood units	8.84 ± 3.83	5.82 ± 2.63	< **0.001**	10.56 ± 3.95	6.22 ± 3.79	< **0.001**
CPET							
	DLCO, % predicted	62.99 ± 14.78	65.87 ± 16.38	**0.003**	58.94 ± 14.97	63.55 ± 14.31	**0.018**
	${\mathrm{FEV}}_{1}$/FVC	0.75 ± 0.07	0.75 ± 0.06	0.925	0.73 ± 0.07	0.74 ± 0.06	**0.016**
	$m_{2}$@Peak, mL/min/kg	12.76 ± 3.88	15.13 ± 3.72	< **0.001**	10.98 ± 1.86	13.28 ± 3.01	< **0.001**
	$P_{\text{ET}}$${\mathrm{CO}}_{2}$@Peak, mmHg	24.54 ± 5.53	29.09 ± 5.33	< **0.001**	23.53 ± 4.59	29.56 ± 5.29	< **0.001**
	VE/${\mathrm{VCO}}_{2}$ slope	48.63 ± 10.56	42.93 ± 8.67	< **0.001**	50.10 ± 7.08	42.57 ± 6.84	< **0.001**

Data are presented as mean ± standard deviation, median (interquartile range) or number (percentage). BPA, balloon pulmonary angioplasty; CPET, cardiopulmonary exercise test; DLCO, diffusing capacity for carbon monoxide; dPAP, diastolic pulmonary arterial pressure; EF, ejection fraction; ${\mathrm{FEV}}_{1}$/FVC, forced expiratory volume in one second/forced vital capacity; LA, anteroposterior diameter of left atrium; mPAP, mean pulmonary arterial pressure; mRAP, mean right atrial pressure; NT-proBNP, N-terminal pro-brain natriuretic peptide; PAWP, pulmonary arterial wedge pressure; $P_{\text{ET}}$${\mathrm{CO}}_{2}$, end-tidal partial pressure of carbon dioxide; PVR, pulmonary vascular resistance; RVED/LVED, right ventricular end-diastolic diameter/ left ventricular end-diastolic diameter; 6MWD, 6-min walk distance; sPAP, systolic pulmonary arterial pressure; $S_{\text{v}}$$O_{2}$, mixed venous oxygen saturation; TRV, tricuspid regurgitation velocity; VE/${\mathrm{VCO}}_{2}$, minute ventilation/carbon dioxide production; ${\mathrm{VO}}_{2}$@Peak, peak oxygen consumption; WHO FC, World Health Organization functional class. Significant *p* values (*p *< 0.05) are bolded.

### 3.5 Impact of BPA-Related Complications on Survival

**Fig. 5.**
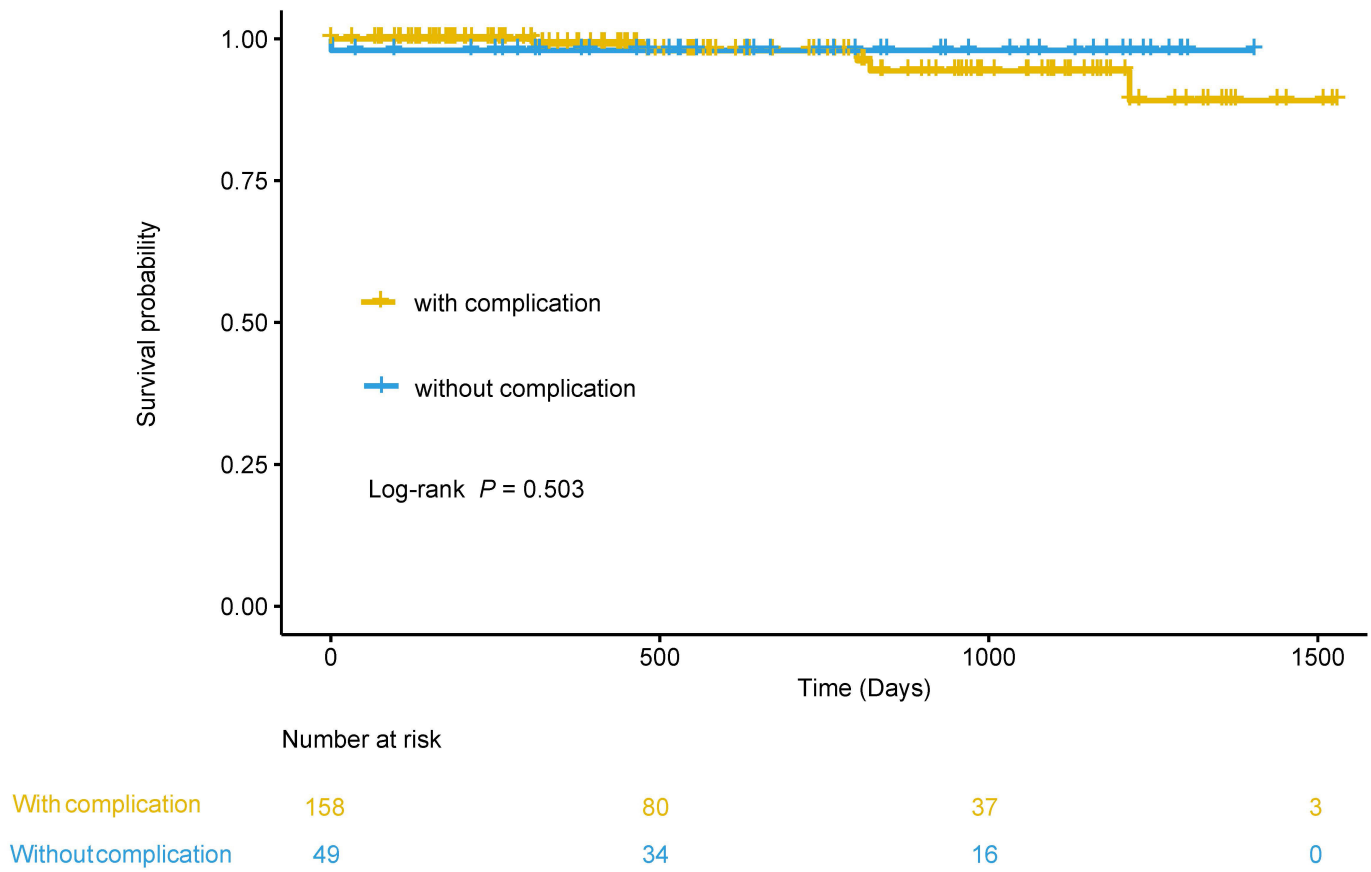
**Kaplan–Meier analysis to compare survival between patients with and without complication**.

After a median of 573 (288.5, 1020) days of follow-up, death occurred in 6 (2.9%) patients. Among them, one (2%) was in patients with BPA-related complications, and 5 (3.2%) were in patients without BPA-related complications. The 1- and 3-year survival rates in the total cohort, patients without complications and patients with complications were 98.9% and 95.5%; 99.1% and 94.3%; 98% and 98%, respectively. The Kaplan–Meier curve illustrated that the survival was comparable between patients with BPA-related complications and those without (log-rank *p* = 0.503) (Fig. 5).

## 4. Discussion

In published articles, several single parameters were identified as predictors of BPA-related complications [8, 9, 10, 13]. In the present study, we constructed a 3-variable nomogram to predict BPA-related complications, which outperformed previously reported predictors [8, 9, 10, 13]. Despite a relatively high incidence, most complications were mild to moderate and could be successfully addressed by balloon occlusion and CPAP. Importantly, the occurrence of BPA-related complications did not impair hemodynamic improvement or long-term survival after BPA.

BPA was initially attempted by Feinstein *et al*. [13] in 2001 among patients with CTEPH. Despite significant hemodynamic improvement, the incidence of reperfusion pulmonary edema was as high as 61%, and 16-month mortality was 11.1%, which discourages many centers from adopting BPA. Subsequently, BPA was abandoned for approximately 10 years. In 2012, Japanese researchers refined the BPA procedure with a smaller balloon and a staged concept, which reduced mortality to 2.94% and demonstrated a significant hemodynamic benefit [14, 15, 16]. Since then, an increasing number of centers have attempted and optimized BPA procedures, reducing the complications from over 60% [16] to 14.5% [8]. In the current study, the incidence of BPA-related complications was 10.26%, which was comparable to previous studies [6, 8]. Most BPA complications identified in the present study were attributable to wire injury of pulmonary arteries, which could be indicated by the following symptoms: oxygen desaturation, hemoptysis or hemosputum or cough without bloody sputum, and extravasation of contrast medium. Mild and moderate pulmonary injuries could be successfully addressed by balloon occlusion and CPAP. In the present study, only one in-hospital death was observed. The patient experienced severe pulmonary reperfusion edema, and CPAP and extracorporeal membrane oxygenation failed to rescue the patient.

### 4.1 Predictors of BPA Complications

Type of lesions is correlated with BPA complications. Compared with nonocclusion lesions, each 1% increase in the proportion of occlusion lesions elevated the risk of complications by 2.7%. Similarly, previous studies have also found that the incidence of complications of subtotal occlusions was as high as 15.5%, whereas that of web lesions was only 2.2% [7]. The underlying mechanism might be attributable to the invisible vessel contour and extremely hard fibrous tissue of occlusion lesions; thus, engaging the guidewire into occluded vessels is extremely challenging. Moreover, when treating occlusion lesions, the tip of the wire has a high risk of entering the subintimal space, causing dissection and vessel injury.

Previous studies have identified that hemodynamics, including baseline mPAP [10, 13] and PVR [8], are predictors of BPA complications, which was also confirmed in the present study. We found that mPAP outperformed PVR in predicting complications. Many complications, especially pulmonary reperfusion injury, are attributable to disproportion between high blood perfusion and vulnerable pulmonary arteries, where the artery bed could not tolerate increased perfusion after BPA. mPAP could reflect the effect of perfusion pressure on pulmonary arteries, while PVR excluded the impact of blood flow, which might account for the superiority of mPAP over PVR.

In addition to hemodynamics, a longer disease duration also contributes to a higher incidence of complications. There is a causal relationship between disease duration and hemodynamics, where the longer the disease duration is, the poorer the hemodynamics.

### 4.2 Impact of Complications on BPA Efficacy

Previous studies have reported that complications could increase the length of hospitalization and medical cost [8]. In the present study, we found that complications did not impair the beneficial effect of BPA on hemodynamics and survival. Patients show significant hemodynamic improvement after BPA, irrespective of whether they experience complications. Moreover, the overall survival was optimal both in patients with and without complications.

## 5. Limitations

The current study had inherent limitations of a retrospective design. Additionally, the study was based on a single center, and only internal validation was performed, which might limit the generalizability of the conclusions and warrants future multicenter studies with external validation. A learning curve is commonly observed in performing BPA. Previous studies demonstrated that accumulated BPA experience contributed to a decreased incidence of complications [9]. The learning curve might bias the study. Therefore, we compared the incidence of complications in the first and second 307 BPA sessions, which was similar [10.42% (32/307) vs. 10.10% (31/307)]. Thus, including all the BPA sessions from different study periods did not bias the conclusion.

## 6. Conclusions

The nomogram, comprising mPAP, occlusion lesions and disease duration, was superior to previously reported single parameters in predicting BPA-related complications. Distinctively, the occurrence of complications did not impair the beneficial impact of BPA on hemodynamics and survival. Patients who experienced complications could also benefit from BPA, and the occurrence of complications should not discourage patients from continuing BPA sessions.

## Data Availability

The data underlying this article will be shared on reasonable request to the corresponding author.

## Abbreviations

AUC, area under the curve; BPA, balloon pulmonary angioplasty; BMI, body mass index; CI, confidence interval; CTEPH, chronic thromboembolic pulmonary hypertension; CPAP, continuous positive airway pressure; DLCO, diffusing capacity for carbon monoxide; dPAP, diastolic pulmonary arterial pressure; EF, ejection fraction; ${\mathrm{FEV}}_{1}$/FVC, forced expiratory volume in one second/forced vital capacity; LA, anteroposterior diameter of left atrium; mPAP, mean pulmonary arterial pressure; mRAP, mean right atrial pressure; NT-proBNP, N-terminal pro-brain natriuretic peptide; PAWP, pulmonary arterial wedge pressure; PEA, pulmonary endarterectomy; $P_{\text{ET}}$${\mathrm{CO}}_{2}$, end-tidal partial pressure of carbon dioxide; PVR, pulmonary vascular resistance; RVED/LVED, right ventricular end-diastolic diameter/left ventricular end-diastolic diameter; 6MWD, 6-min walk distance; sPAP, systolic pulmonary arterial pressure; $S_{\text{v}}$$O_{2}$, mixed venous oxygen saturation; TRV, tricuspid regurgitation velocity; PH, pulmonary hypertension; PVR, pulmonary vascular resistance; RHC, right heart catheterization; ROC, receiver operator characteristic curve; VE/${\mathrm{VCO}}_{2}$, minute ventilation/carbon dioxide production; ${\mathrm{VO}}_{2}$, oxygen uptake; WHO-FC, World Health Organization functional class.

## Supplementary Material

Supplementary material associated with this article can be found, in the online version, at https://doi.org/10.31083/j.rcm2403072.
