# Supplementary material for: Development and Validation of a Nomogram for Balloon Pulmonary Angioplasty-Related Complications in Patients with Chronic Thromboembolic Pulmonary Hypertension
Source: Rev Cardiovasc Med. 2023 Feb 28;24(3):72. doi: 10.31083/j.rcm2403072 (PMC11263984; doi:10.31083/j.rcm2403072)
Supplement: Supplementary file 1 [file 2153-8174-24-3-072-s1.docx]

**Supplementary Methods:**

Before each balloon pulmonary angioplasty (BPA) session, right heart catheterization was performed to obtain the hemodynamic parameters, including the right atrial and ventricular pressure, mean pulmonary arterial pressure (mPAP), pulmonary arterial wedge pressure, cardiac output (calculated by indirect Fick’s method) and oxygen saturation. Mixed venous oxygen saturation (S_v_O_2_) and pulmonary vascular resistance (PVR) were calculated in line with standard equations[^7^](#_ENREF_7). A 6 Fr guiding catheter (Multipurpose [Cordis Corporation, Bridgewater, New Jersey, USA], Amplatz left catheter [Terumo® Heartrail™ II; Terumo Corporation, Tokyo, Japan] or Judkins right catheter [Terumo® Heartrail™ II; Terumo Corporation, Tokyo, Japan]) was advanced to pulmonary arteries via a 7 Fr long sheath (Flexor® Check-Flo® Introducer; Cook Medical, Bloomington, IN, USA). With selective pulmonary angiography as reference, we inserted a 0.014-inch guidewire (e.g. Hi-Torque Pilot 50; Abbot, Santa Clara, CA, USA) across the target pulmonary lesion, and then inflated the balloon to dilate the target pulmonary lesions. In initial dilation, a smaller balloon was initially used to avoid complications. In the following dilations, the inflation pressure and size of balloon were dynamically adjusted in accordance with targeted vascular diameter and vascular response. Hemodynamic parameters were measured again after each BPA session.

# Supplementary Table 1. Change of parameters of patients with/ without complications

| Variables | Without complication  (n=115) | With complication  (n=45) | *P* value |
| --- | --- | --- | --- |
| ΔNT-proBNP, ng/L | –367.00 (–1056.50, –44.60) | –502.00 (–1113.30, –40.35) | 0.692 |
| Δ6MWD, m | 61.00 (16.00, 111.50) | 90.00 (36.00, 175.00) | 0.051 |
| Echocardiography |  |  |  |
| Δ LA, mm | 1.00 (–2.00, 3.00) | 2.00 (–1.00, 4.50) | 0.276 |
| ΔRVED/LVED | –0.10 (–0.20, 0.00) | –0.16 (–0.30, –0.04) | 0.125 |
| ΔEF, % | 2.00 (–2.00, 5.00) | 0.00 (–5.00, 6.00) | 0.253 |
| Hemodynamics |  |  |  |
| ΔmRAP, mmHg | 0.00 (–3.00, 2.00) | 0.00 (–3.50, 2.00) | 0.679 |
| ΔsPAP, mmHg | –16.00 (–35.00, –6.00) | –23.00 (–46.00, –9.50) | 0.173 |
| ΔdPAP, mmHg | –6.00 (–10.00, –1.00) | –7.00 (–15.00, –3.00) | 0.169 |
| ΔmPAP, mmHg | –10.00 (–17.00, –3.00) | –12.00 (–23.00, –5.50) | 0.115 |
| ΔCardiac index, L/(min·m^2^) | 0.32 (–0.04, 0.73) | 0.57 (–0.03, 1.28) | 0.061 |
| ΔPVR, wood units | –2.30 (–4.94, –0.81) | –4.37 (–6.15, –1.72) | **0.021** |

Data are presented as mean ± standard deviation, median (interquartile range) or number (percentage). BPA, balloon pulmonary angioplasty; dPAP, diastolic pulmonary arterial pressure; EF, ejection fraction; LA, left atrium dimension;, mPAP, mean pulmonary arterial pressure; mRAP, mean right atrial pressure; NT-proBNP, N-terminal pro-brain natriuretic peptide; PVR, pulmonary vascular resistance; RVED/ LVED, right ventricular end-diastolic diameter/ left ventricular end-diastolic diameter; 6MWD, 6-min walk distance; sPAP, systolic pulmonary arterial pressure; change of parameters was calculated by parameters at follow-up minus parameters at baseline.

# Supplementary Table 2. Multivariable linear regression analysis for change of parameters after BPA.

| Variables | β | 95 % Lower Bound | 95% Upper Bound | *P* |
| --- | --- | --- | --- | --- |
| Response: change of cardiac index |  |  |  |  |
| Complication | 0.249 | –0.006 | 0.504 | 0.055 |
| BPA session | 0.005 | –0.070 | 0.079 | 0.904 |
| Response: change of NT–proBNP |  |  |  |  |
| Complication | –47.443 | –396.392 | 301.505 | 0.789 |
| BPA session | –73.156 | –175.001 | 28.689 | 0.158 |
| Response: change of mPAP |  |  |  |  |
| Complication | –0.826 | –4.299 | 2.648 | 0.639 |
| BPA session | –3.254 | –4.268 | –2.240 | **<0.001** |
| Response: change of PVR |  |  |  |  |
| Complication | –0.827 | –2.012 | 0.359 | 0.170 |
| BPA session | –0.503 | –0.849 | –0.157 | **0.005** |

BPA, balloon pulmonary angioplasty; NT-proBNP, N-terminal pro-brain natriuretic peptide; *for each increase of 1000 ng/L in NT-proBNP
